# Prediction of COVID-19 Patients at High Risk of Progression to Severe Disease

**DOI:** 10.3389/fpubh.2020.574915

**Published:** 2020-11-24

**Authors:** Zhenyu Dai, Dong Zeng, Dawei Cui, Dawei Wang, Yanling Feng, Yuhan Shi, Liangping Zhao, Jingjing Xu, Wenjuan Guo, Yuexiang Yang, Xinguo Zhao, Duoduo Li, Ye Zheng, Ao Wang, Minmin Wu, Shu Song, Hongzhou Lu

**Affiliations:** ^1^Department of Invasive Technology, Yancheng Clinical Medical College of Nanjing Medical University, Yancheng, China; ^2^Department of Pathology, Shanghai Public Health Clinical Center, Fudan University, Shanghai, China; ^3^Department of Blood Transfusion, The First Affiliated Hospital, Zhejiang University School of Medicine, Hangzhou, China; ^4^Department of Infectious Disease, The Second People's Hospital of Yancheng City, Yancheng, China; ^5^Department of Gynecology and Obstetrics, Tongji Medical College, Wuhan Central Hospital, Huazhong University of Science and Technology, Wuhan, China; ^6^Department of Respiration, The Fifth People's Hospital of Wuxi, Wuxi, China; ^7^Department of Infectious Disease and Immunology, Shanghai Public Health Clinical Center, Fudan University, Shanghai, China

**Keywords:** COVID-19, severity, risk factors, scoring model, nomogram

## Abstract

In order to develop a novel scoring model for the prediction of coronavirus disease-19 (COVID-19) patients at high risk of severe disease, we retrospectively studied 419 patients from five hospitals in Shanghai, Hubei, and Jiangsu Provinces from January 22 to March 30, 2020. Multivariate Cox regression and orthogonal projections to latent structures discriminant analysis (OPLS-DA) were both used to identify high-risk factors for disease severity in COVID-19 patients. The prediction model was developed based on four high-risk factors. Multivariate analysis showed that comorbidity [hazard ratio (HR) 3.17, 95% confidence interval (CI) 1.96–5.11], albumin (ALB) level (HR 3.67, 95% CI 1.91–7.02), C-reactive protein (CRP) level (HR 3.16, 95% CI 1.68–5.96), and age ≥60 years (HR 2.31, 95% CI 1.43–3.73) were independent risk factors for disease severity in COVID-19 patients. OPLS-DA identified that the top five influencing parameters for COVID-19 severity were CRP, ALB, age ≥60 years, comorbidity, and lactate dehydrogenase (LDH) level. When incorporating the above four factors, the nomogram had a good concordance index of 0.86 (95% CI 0.83–0.89) and had an optimal agreement between the predictive nomogram and the actual observation with a slope of 0.95 (*R*^2^ = 0.89) in the 7-day prediction and 0.96 (*R*^2^ = 0.92) in the 14-day prediction after 1,000 bootstrap sampling. The area under the receiver operating characteristic curve of the COVID-19-American Association for Clinical Chemistry (AACC) model was 0.85 (95% CI 0.81–0.90). According to the probability of severity, the model divided the patients into three groups: low risk, intermediate risk, and high risk. The COVID-19-AACC model is an effective method for clinicians to screen patients at high risk of severe disease.

## Highlights

- The severity and mortality of COVID-19 patients urgently need to be resolved.
- Comorbidity, ALB, CRP, and age ≥60 years are independent risk factors for severe COVID-19.
- The COVID-19-AACC model is effective for screening patients at risk of severe disease.

## Introduction

In December 2019, an increasing number of patients with pneumonia of unknown cause were found in Wuhan, China (1, 2). A novel coronavirus was identified by gene detection and virus isolation. On January 12, 2020, the World Health Organization (WHO) named the virus “2019-nCoV” (3), and on February 11, 2020, the WHO renamed it severe acute respiratory syndrome coronavirus 2 (SARS-CoV-2) and the disease it caused coronavirus disease 2019 (COVID-19) (4). The epidemic soon spread all over China and 212 other countries and areas around the world, resulting in more than 4.72 million people infected and over 300,000 deaths up to May 17, 2020. It has been shown that COVID-19 is more contagious than SARS-CoV seen in 2003, and that medical staff were also infected during the epidemic (5, 6).

Wu et al. (7) first reported that timely antiviral treatment may slow the progression of COVID-19 caused by SARS-CoV-2 and improve the prognosis. Nahama et al. (8) found that the use of resiniferatoxin could improve patient outcomes in those with advanced COVID-19. Omarjee et al. (9) demonstrated that targeting T-cell senescence and cytokine storm with rapamycin may prevent progression in COVID-19. However, up to the date of submission of this report, there are still no specific drugs for COVID-19 patients worldwide, and the severity and mortality of COVID-19 patients are urgent problems that still need to be resolved (10, 11). Hence, it is extremely important to understand the critical factors associated with the severity of COVID-19 and provide convenient and efficient diagnostic methods. Xiao et al. (12) developed an artificial intelligence-assisted tool using computed tomography (CT) imaging to predict disease severity and further estimate the risk of developing severe disease in patients suffering from COVID-19.

In the present study, we aimed to develop a novel scoring model for predicting patients at high risk of severe COVID-19, which would facilitate clinicians to manage COVID-19 patients.

## Patients and Methods

### Patients

In this study, 419 consecutive patients with confirmed COVID-19 were enrolled from the Shanghai Public Health Clinical Center (208 cases), the Wuhan Central Hospital, Tongji Medical College, Huazhong University of Science and Technology (130 cases), the third People's Hospital of Yancheng City (15 cases), the Fifth People's Hospital of Wuxi (46 cases), and the Second People's Hospital of Yancheng City (20 cases) from January 22 to March 30, 2020, and the follow-up ended on April 30, 2020. All patients admitted with severe COVID-19 to these five hospitals were excluded. This retrospective study was performed in accordance with the Helsinki Declaration and was approved by the Ethics Committee of the Shanghai Public Health Clinical Center (YJ-2020-S089-02).

### Definition and Clinical Classification of Cases

All the enrolled COVID-19 patients were diagnosed based on the WHO criteria (13) and the National Health Commission of China criteria. We defined the COVID-19 patients according to epidemiological history consistent with any two clinical manifestations and pathogenic evidence. SARS-CoV-2 RNA was tested with samples from the nose, pharynx, and anus swabs, respectively, by real time-polymerase chain reaction (PCR). We defined the clinical classification and epidemiological history of COVID-19 patients as described previously (14): the first generation (Generation I): patients with a history of exposure to the south China seafood market in Wuhan, China; the second generation (Generation II): patients with Wuhan tourism experience; the third generation (Generation III): imported cases; and the fourth generation (Generation IV): patients infected by Generation III patients. The progression to severe COVID-19 during the observation period was diagnosed based on heart and pulmonary function recovery and lung CT findings. We divided the patients into the severe group and the stable group according to whether the patients had progression to severe COVID-19. In this study, all COVID-19 patients who also had other virus infections were excluded.

### Data Collection

We retrospectively collected data from the patients' medical records and attending doctors, including clinical baseline data, laboratory parameters, length of stay, and so on. At the time of admission, all patients underwent laboratory examinations. All data were collected on the first day after admission. Clinical outcomes were followed up till April 30, 2020.

### Statistical Analysis

Statistical analyses were performed by SPSS (version 25; IBM SPSS Statistics, United States) and R software, version 3.6.1 (R Foundation for Statistical Computing, Vienna, Austria). Continuous variables with normal distribution were expressed as mean ± standard deviation and were compared using the independent sample *t*-test. Data with non-normal distribution were expressed as median (IQR) and were compared using the non-parametric test. The classified variables were tested using the chi-square test. A value of *P* < 0.05 was considered statistically significant. The significance of each variable was assessed using the univariate and multivariate Cox proportional hazards model to investigate the independent high-risk factors for disease severity with their hazard ratio (HR) and 95% confidence interval (CI). The performance of the nomogram was evaluated by calibration with 1,000 bootstrap samples to decrease the overfit bias. The receiver operating characteristic (ROC) package in R software was used to compare the time-dependent area under the ROC curve (td-AUC). Orthogonal projections to latent structures discriminant analysis (OPLS-DA) was performed with SIMCA version 14.1.0.2047.

## Results

### Clinical Characteristics of COVID-19 Patients in the Severe Group and the Stable Group

**Figure 1:**
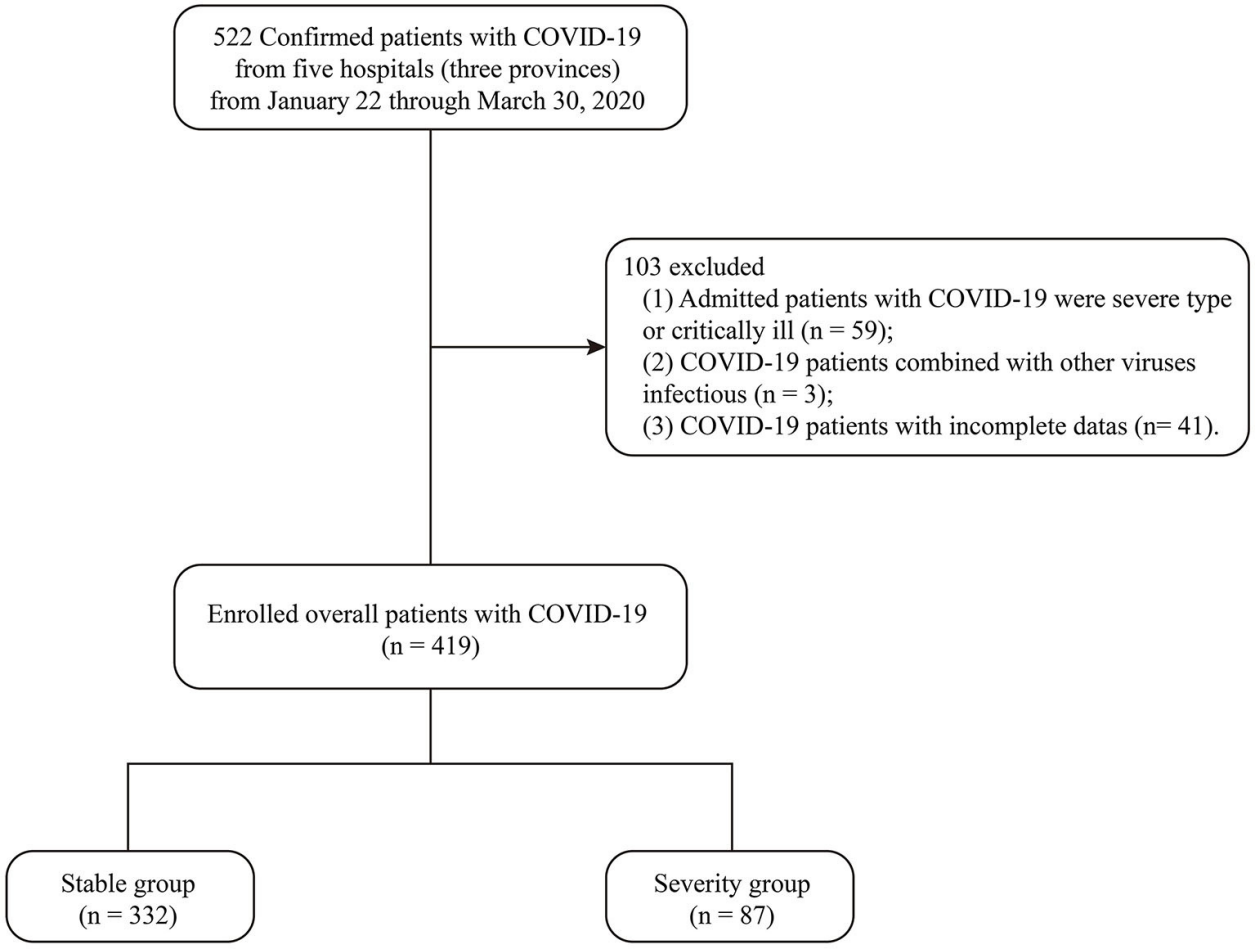
Flowchart of COVID-19 patient enrollment including the stable group and the severe group.

A total of 419 eligible COVID-19 patients included in the stable group and the severe group were recruited from five hospitals in Shanghai, Jiangsu, and Hubei Provinces, China. The flowchart of patient enrollment is shown in Figure 1. The clinical characteristics of these patients are summarized in Table 1. In these 419 patients, the average age was 47.1 ± 16.0 years, 207 (49.4%) were male, 88 (21.0%) were older than 60 years, 139 (33.2%) had at least one underlying comorbidity, the average hospitalization time was 16.5 ± 7.9 days, and 87 (20.8%) patients became severe and critically ill during the observation period. Three hundred fourteen (74.9%) patients were Generations III and IV.

**Table 1:** Characteristics of COVID-19 patients in this study.

	**Overall**	**Stable group**	**Severity group**	***P* value**
	**(*****n*** **= 419)**	**(*****n*** **= 332)**	**(*****n*** **= 87)**	
Age, years	47.1 ± 16.0	44.6 ± 15.2	56.8 ± 15.4	0.000
Gender (n, %)				0.053
Male	207 (49.40)	156 (46.99)	51 (58.62)	
Female	212 (50.60)	176 (53.01)	36 (41.38)	
Comorbidity (n, %)				0.000
Without	280 (66.83)	249 (75.00)	31 (35.63)	
With	139 (33.17)	83 (25.00)	56 (64.37)	
Lymphocyte, × 10^9^/L	1.2 (0.8–1.6)	1.2 (0.9–1.7)	0.8 (0.6–1.1)	0.000
D-dimer, mg/L	0.28 (0.19–0.54)	0.24 (0.17–0.45)	0.51 (0.30–0.91)	0.000
ALT, U/L	24 (15–38)	23 (14–38)	27 (18–39)	0.072
ALB	40 (37–43)	41 (38–44)	37 (33–39)	0.000
TBIL, μmol/L	10.4 (7.3–15.4)	9.8 (7.0–14.5)	13.4 (8.3–18.8)	0.002
LDH, U/L	213 (172–281)	204 (166–256)	281 (212–346)	0.000
CRP	8.0 (2.6–26.3)	6.2 (1.8–18.4)	39.0 (15.0–85.2)	0.000
PCT, μg/L	0.05 (0.02–0.05)	0.04 (0.02–0.05)	0.05 (0.04–0.09)	0.000
D-dimer, mg/L				0.000
≤0.55	318 (75.89)	269 (81.02)	49 (56.32)	
>0.55	101 (24.11)	63 (18.98)	38 (43.68)	
Lymphocyte, × 10^9^/L (n, %)				0.000
>1.0	259 (61.81)	230 (69.28)	29 (33.33)	
≤1.0	160 (38.19)	102 (30.72)	58 (66.67)	
Age, years (n, %)				0.000
≤60	331 (79.00)	287 (86.45)	44 (50.57)	
>60	88 (21.00)	45 (13.55)	43 (49.43)	
LDH, U/L (n, %)				0.000
≤250	272 (64.92)	241 (72.59)	31 (35.63)	
250–500	138 (32.94)	86 (25.90)	52 (59.77)	
>500	9 (2.15)	5 (1.51)	4 (4.60)	
CRP, mg/L (n, %)				0.000
<10	229 (54.65)	213 (64.16)	16 (18.39)	
≥10	190 (45.35)	119 (35.84)	71 (85.06)	
ALB, g/L (n, %)				0.000
≥40	215 (51.31)	202 (60.84)	13 (14.94)	
<40	204 (48.69)	130 (39.16)	74 (85.06)	

**Figure 2:**
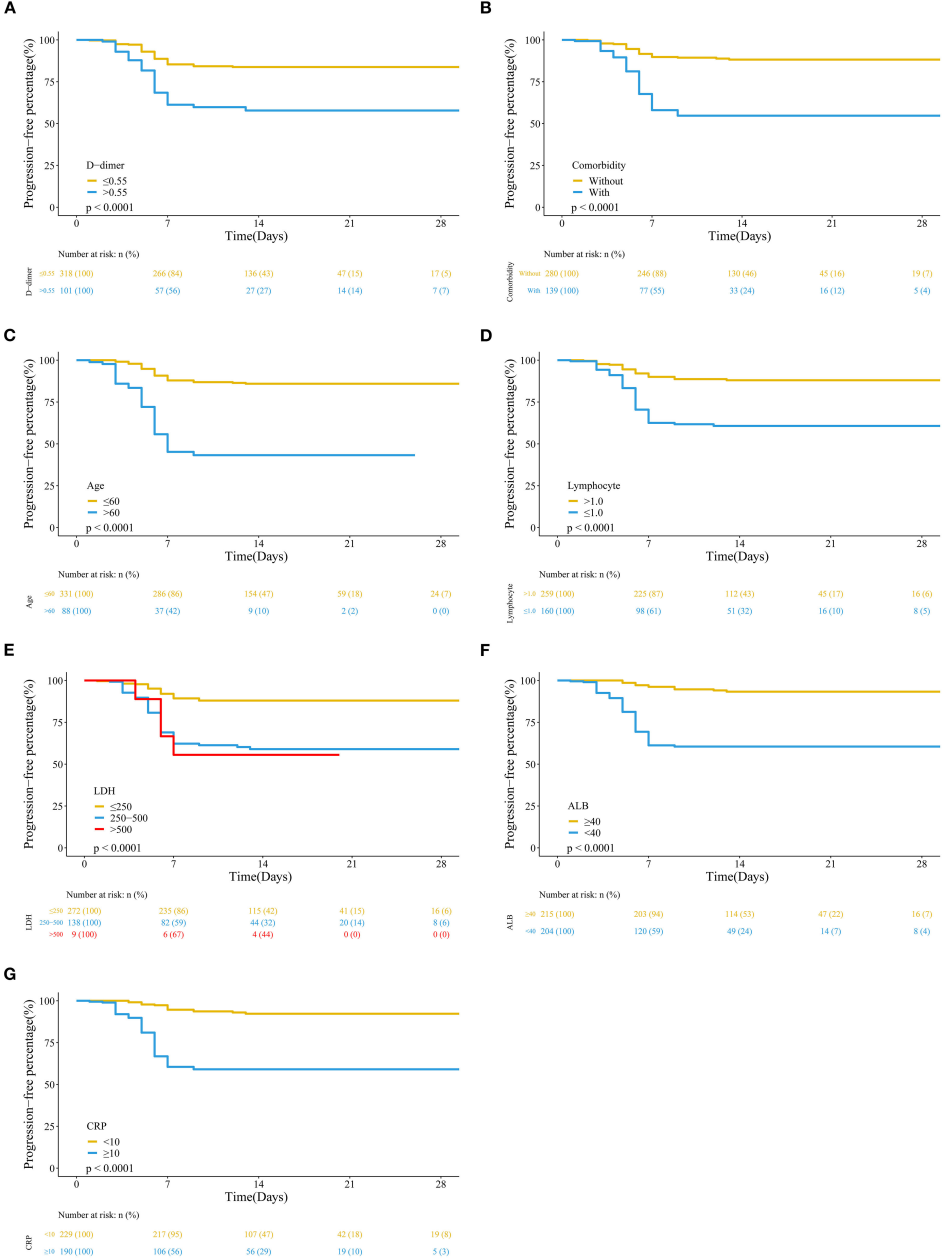
Kaplan–Meier analysis of high-risk factors for severe COVID-19. We defined the time from admission after infection and days to severe disease development or discharge. **(A)** D-dimer; **(B)** comorbidity; **(C)** age; **(D)** lymphocyte count; **(E)** lactate dehydrogenase (LDH); **(F)** albumin (ALB); **(G)** C-reactive protein (CRP).

When the clinical characteristics in the stable group and the severe group were compared, the results showed that age, comorbidity, lymphocyte count, albumin (ALB), D-dimer, C-reactive protein (CRP), and lactate dehydrogenase (LDH) levels were significantly different between the two groups (Table 1 and Figure 2).

### High-Risk Factors for Severe COVID-19

We performed Cox regression analysis, which demonstrated that comorbidity (HR 3.17, 95% CI 1.96–5.11), ALB level (HR 3.67, 95% CI 1.91–7.02), CRP level (HR 3.16, 95% CI 1.68–5.96), and age ≥60 years (HR 2.31, 95% CI 1.43–3.73) were independent risk factors for severe COVID-19 in these patients (Table 2).

**Table 2:** The univariate and multivariate logistic regression analysis independent high-risk factors for severity of COVID-19 patients.

	**Un COX analysis**		**Mul COX analysis**	
	**HR (95% CI)**	***P*** **value**	**HR (95% CI)**	***P*** **value**
D-dimer (mg/L)				
≤0.55	1	–	1	–
>0.55	2.961 (1.936–4.529)	0.000	1.070 (0.672–1.702)	0.776
Comorbidity				
Without	1	–	1	–
With	4.617 (2.971–7.173)	0.000	3.166 (1.960–5.114)	0.000
Age (years)				
≤60	1	–	1	–
>60	5.557 (3.633–8.499)	0.000	2.307 (1.427–3.728)	0.001
Lymphocyte (×10/L)				
>1.0	1	–	1	–
≤1.0	3.814 (2.440–5.961)	0.000	1.234 (0.741–2.054)	0.419
LDH (U/L)				
≤250	1	–	1	–
250–500	3.944 (2.526–6.158)	0.000	1.531 (0.918–2.553)	0.103
>500	4.215 (1.487–11.943)	0.007	2.572 (0.869–7.612)	0.088
ALB (g/L)				
≥40	1	–	1	–
<40	7.899 (4.374–14.267)	0.000	3.663 (1.912–7.018)	0.000
CRP (mg/L)				
<10	1	–	1	–
≥10	7.022 (4.076–12.098)	0.000	3.161 (1.677–5.961)	0.000

**Figure 3:**
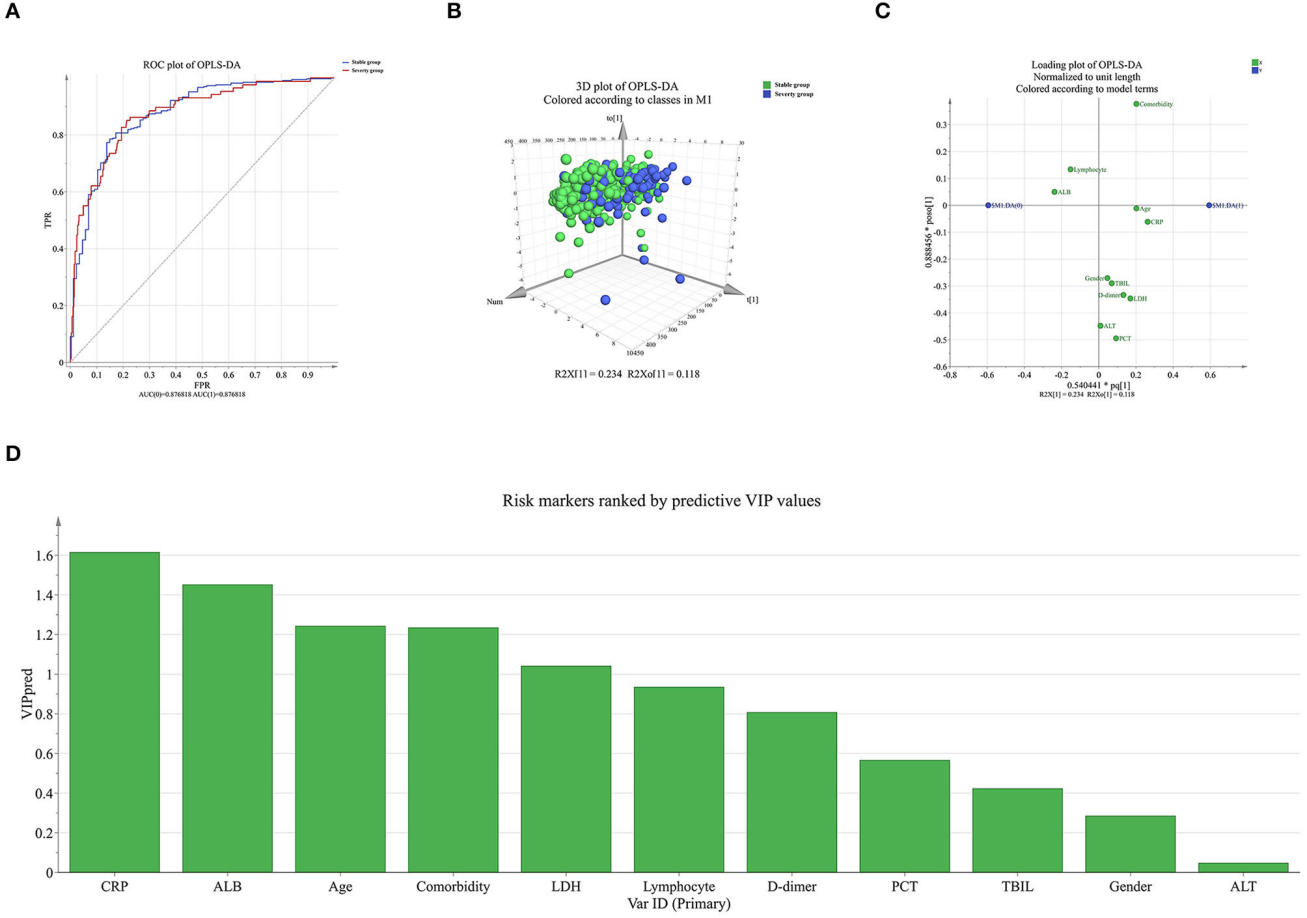
OPLS-DA to evaluate the influence of parameters on the severity of COVID-19. **(A)** ROC of OPLS-DA. **(B)** In the three-dimensional scatter plot of all samples in the OPLS-DA model, the predictive component was used in the stable group and the severe group. **(C)** Loading plot showing the relationship of each parameter to the predictive component (x) and the first orthogonal component (y); parameters that deviated from zero on the x-axis were considered potentially predictive. **(D)** The higher predictive VIP (VIP pred) value.

We also used OPLS-DA to evaluate the influence of parameters on the severity of COVID-19. The severe group was unambiguously distinguished from the stable group (Figures 3A,B). The top five parameters that influenced the severity of COVID-19 were CRP, ALB, age ≥60 years, comorbidity, and LDH (Figures 3C,D).

Hence, comorbidity, ALB, CRP, and age ≥60 years were identified as the most influential risk factors for the severity of COVID-19 in these patients.

### Development and Validation of a Predictive Nomogram for the Probability of Severe COVID-19

**Figure 4:**
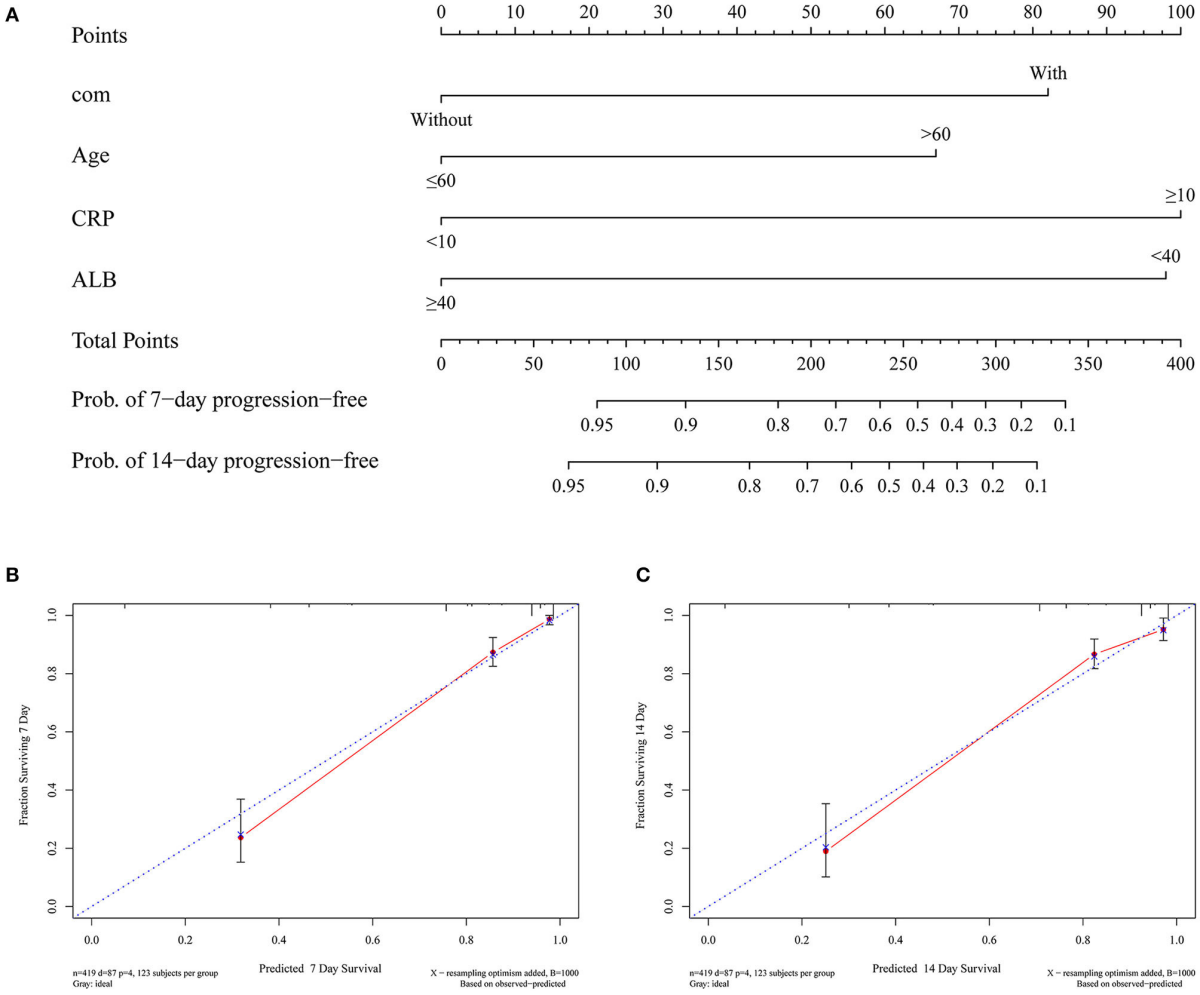
Development and validation of a predictive nomogram for the probability of severe COVID-19. **(A)** A predictive nomogram was developed based on the independent risk factors associated with the severity of COVID-19. **(B)** The 7-day predictive performance of the nomogram with a slope of 0.95 (*R*^2^ = 0.89). **(C)** The 7-day predictive performance of the nomogram with a slope of 0.96 (*R*^2^ = 0.92).

Based on the above independent risk factors associated with the severity of COVID-19, we developed a predictive nomogram and validated it using the bootstrap method (Figure 4A). Calibration tests were used to evaluate the predictive accuracy for progression of COVID-19 using the nomogram. The C-index for predicting the severity of COVID-19 with the nomogram was 0.86 (0.83–0.89), which indicated good accuracy. The calibration curve showed optimal agreement between the predictive nomogram and the actual observation with a slope of 0.95 (*R*^2^ = 0.89) in the 7-day prediction and 0.96 (*R*^2^ = 0.92) in the 14-day prediction after 1,000 bootstrap sampling (Figures 4B,C).

### Development and Assessment of the Novel Scoring Model for COVID-19 Severity

**Figure 5:**
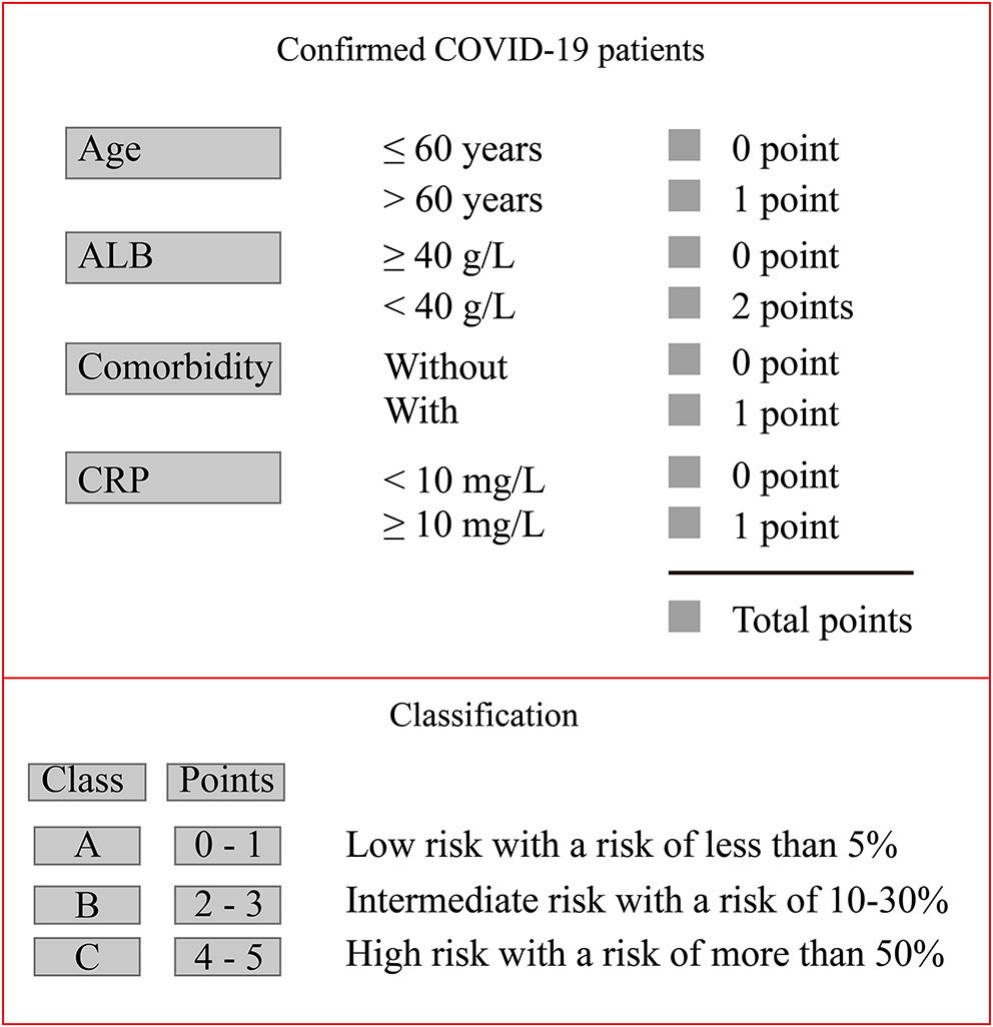
COVID-19-AACC model for risk stratification of the probability of severe COVID-19.

Based on the above nomogram, we further developed a novel scoring model, which may facilitate the clinical assessment of COVID-19 severity. We named the model COVID-19-American Association for Clinical Chemistry (AACC) (age ≥60 years, ALB, comorbidity, and CRP), and the score ranged from 0 to 5 points (Figure 5). CRP (<10 mg/L) and ALB (<40 g/L) were chosen as the cut-off values, respectively, to score the ALB and CRP.

The AUROC of the COVID-19-AACC scoring model for predicting the probability of severe COVID-19 in these patients was 0.85 (95% CI 0.81–0.90).

With the cut-off value of 0 point, the positive predictive value and positive likelihood ratio of the scoring model were 27.2 (95% CI 22.3–32.5) and 1.42 (95% CI 1.3–1.5). The negative predictive value and negative likelihood ratio were 97.3 (95% CI 92.2–99.4) and 0.11 (95% CI 0.03–0.3), with a sensitivity of 96.6 (95% CI 90.3–99.3) and a specificity of 32.2 (95% CI 27.2–37.5).

Using a cut-off value of 4 points, the positive predictive value and positive likelihood ratio of the scoring model were 80.6 (95% CI 62.5–92.5) and 15.90 (95% CI 6.7–37.5). The negative predictive value and negative likelihood ratio were 84.0 (95% CI 80.0–87.5) and 0.73 (95% CI 0.6–0.8), with a sensitivity of 28.7 (95% CI 19.5–39.4) and a specificity of 98.2 (95% CI 96.1–99.3) (Table 3).

**Table 3:** The performances of COVID-19-AACC model for risk stratification of probabilities for severity of COVID-19 patients.

**Variable**	**(*n* = 419)**
AUROC	0.85 (0.81–0.90)
Cutoff value (95% CI)	0
Sensitivity, %	96.6 (90.3–99.3)
Specificity, %	32.2 (27.2–37.5)
Positive predictive value, %	27.2 (22.3–32.5)
Negative predictive value, %	97.3 (92.2–99.4)
Positive likelihood ratio	1.42 (1.3–1.5)
Negative likelihood ratio	0.11 (0.03–0.3)
Cutoff value (95% CI)	4
Sensitivity, %	28.7 (19.5–39.4)
Specificity, %	98.2 (96.1–99.3)
Positive predictive value, %	80.6 (62.5–92.5)
Negative predictive value, %	84.0 (80.0–87.5)
Positive likelihood ratio	15.90 (6.7–37.5)
Negative likelihood ratio	0.73 (0.6–0.8)

The following three risk groups according to their probability of severe COVID-19 were developed: low risk (Class A: 0–1 point) group, with a risk of severe disease of <5%; intermediate risk (Class B: 2–3 points) group, with a risk of 10–30%; and high-risk (Class C: 4–5 points) group, with a risk of more than 50% (Figure 5).

## Discussion

Coronavirus is distributed throughout the world and has many subtypes. SARS in 2003 and Middle East respiratory syndrome (MERS) in 2013 were caused by coronavirus infection (15, 16). At present, the rapid spread of SARS-CoV-2 worldwide has resulted in a heavy burden to society. To date, the global control of COVID-19 was still not optimistic (17, 18). Although the overall mortality of COVID-19 is not high internationally, the mortality of patients with severe and critical disease is relatively high (19). According to the WHO, the death rate in critically ill patients was over 50% (20). Obviously, it is extremely important to manage these serious cases in a timely and appropriate manner. In fact, in the majority of regions and countries, rapid diagnosis of suspected cases has been possible (21). Thus, how to control the progression from mild to severe disease in these patients is the key to the treatment of COVID-19 by clinicians.

In view of this issue, several studies (22, 23) have shown the factors that may affect the severity of COVID-19. Ji et al. (24) showed that comorbidity, older age, lower lymphocyte count, and higher LDH level were associated with the progression of COVID-19. Yan et al. (25) described the clinical and laboratory characteristics of 193 patients with severe COVID-19. Of these patients, 48 with severe COVID-19 had diabetes. Diabetes was associated with an increased risk of death. Another study (26) showed that severe CO_2_ retention and acidosis prior to extracorporeal membrane oxygenation were confirmed to be risk factors for severe COVID-19 and poor prognosis.

In this study, we retrospectively studied 419 patients from five hospitals in Shanghai, Hubei, and Jiangsu Provinces and determined several risk factors for the severity of COVID-19 in these patients, including age ≥60 years, ALB level, comorbidity, and CRP level. Of the 419 enrolled cases, both median age and the proportion of patients over 60 years in the severe COVID-19 group were significantly higher than those in the stable group (Table 1). The above conclusions were consistent with most previous studies, such as those by Wang et al. (27). It is notable that patients with comorbidities, especially diabetes and cardiovascular diseases, were prone to severe COVID-19. Ji et al. (24) showed that comorbidity, older age, lower lymphocyte count, and higher LDH level at presentation were independent high-risk factors for COVID-19 progression. Zhang et al. (28) selected risk factors for severe and even fatal pneumonia and created a predictive scoring system, including age, white blood cell count, neutrophil count, glomerular filtration rate, and myoglobin level as candidates for the scoring system to predict the severity of COVID-19. We also considered the reasons for the decline in physical function and immune function in the elderly, which could increase the probability of severe COVID-19. The study by Cai et al. (29) indicated that CRP, procalcitonin (PCT), and D-dimer may predict the severity of COVID-19. The study by Zhou et al. (30) showed no significant differences in CRP between the non-aggravation group and the aggravation group. In our study, the levels of CRP in the severe group were significantly higher than those in the stable group, and the proportion of patients with CRP levels ≥10 mg/L was also significantly higher than that in the stable group. Mishra et al. (31) recommended serum ALB for the therapy of SARS-CoV-2. Bi et al. (32) showed that ALB was much lower in severe patients, but was not an independent risk factor for disease progression. Our study has confirmed that ALB is a risk factor for the severity of COVID-19. In the present study, we also assessed the critical factors for disease severity using logistic analysis and OPLS-DA, respectively. The results of both analyses showed that comorbidity, ALB, CRP, and age ≥60 years were the most influential risk factors for severe COVID-19 in these patients.

Based on the above risk factors, we developed a predictive nomogram for the probability of severe COVID-19. The nomogram had a good concordance index of 0.86 (95% CI 0.83–0.89) and well-fitted calibration curves in both the 7-day prediction and the 14-day prediction. We then constructed a scoring model (COVID-19-AACC) based on the above nomogram, which also had a good concordance index of 0.85 (95% CI 0.81–0.90). The COVID-19-AACC scoring model was used to identify COVID-19 patients at low risk (Class A), intermediate risk (Class B), and high risk (Class C) of severe disease. Of the 419 patients enrolled, 254 (60.6%) scored 0–1 point and were considered low risk, and 134 (32.0%) scored 2–3 points and were considered intermediate risk, whereas 31 (7.4) scored 4–5 points and were considered high risk. These high-risk patients should be transferred to tertiary centers as early as possible for appropriate treatment.

Of note, there were several limitations in the present study. Firstly, this study is a retrospective, multicenter study, and the possibility of recall bias cannot be completely excluded. The results from a limited sample size do not necessarily represent the overall results of patients in China or even in the world. Secondly, a validation group should be included to further validate the scoring model. Finally, more indicators, including genes and images, should be included to further optimize the model.

In summary, the COVID-19-AACC scoring model will be of significant help to clinicians in evaluating COVID-19 patients in the early stage, especially in non-tertiary hospitals. For high-risk groups, early intervention can effectively reduce the rate of severe disease and mortality.

## Data Availability Statement

The original contributions presented in the study are included in the article/supplementary materials, further inquiries can be directed to the corresponding author/s.

## Ethics Statement

The studies involving human participants were reviewed and approved by the present retrospective study was performed in accordance with the Helsinki Declaration and was approved by the Ethics Committee of the Shanghai Public Health Clinical Center (YJ-2020-S089-02). The patients/participants provided their written informed consent to participate in this study. Written informed consent was obtained from the individual(s) for the publication of any potentially identifiable images or data included in this article.

## Author Contributions

ZD, DC, DW, and DZ contributed to the study concept and design, conducted the literature search, and wrote the manuscript. YF, JX, WG, and YY contributed to the data analysis and produced the tables and figures. YS, LZ, and XZ contributed to the collection of patient samples and medical information. JX and SS obtained funding. DL, YZ, MW, and AW contributed to the acquisition and analysis of data. HL and SS contributed to the study concept and critically revised the manuscript. All authors contributed to the article and approved the submitted version.

## Conflict of Interest

The authors declare that the research was conducted in the absence of any commercial or financial relationships that could be construed as a potential conflict of interest.

## Abbreviations

COVID-19: coronavirus disease-19
SARS-CoV-2: severe acute respiratory syndrome coronavirus 2
SARS: severe acute respiratory syndrome
MERS: Middle East respiratory syndrome
WHO: World Health Organization
HR: hazard ratio
CI: confidence interval
OPLS-DA: orthogonal projections to latent structures discriminant analysis
CRP: C-reactive protein
ALB: albumin
LDH: lactate dehydrogenase.
